# Prevalence and Trajectory of COVID-19-Associated Hypercoagulability Using Serial Thromboelastography in a South African Population

**DOI:** 10.1155/2021/3935098

**Published:** 2021-12-22

**Authors:** Sarah Alexandra van Blydenstein, Colin Nigel Menezes, Nicole Miller, Naomi Johnson, Bavinash Pillay, Barry F. Jacobson, Shahed Omar

**Affiliations:** ^1^Division of Pulmonology, Department of Internal Medicine, Chris Hani Baragwanath Academic Hospital, University of the Witwatersrand, Johannesburg, South Africa; ^2^Division of Infectious Diseases, Department of Internal Medicine, Chris Hani Baragwanath Academic Hospital, University of the Witwatersrand, Johannesburg, South Africa; ^3^Department of Internal Medicine, Chris Hani Baragwanath Academic Hospital, University of the Witwatersrand, Johannesburg, South Africa; ^4^Department of Molecular Medicine and Haematology, National Health Laboratory Service, University of the Witwatersrand, Johannesburg, South Africa; ^5^Department of Critical Care, Chris Hani Baragwanath Academic Hospital, University of the Witwatersrand, Johannesburg, South Africa

## Abstract

**Introduction:**

The coagulation abnormalities resulting from severe acute respiratory syndrome coronavirus 2 (SARS-CoV-2) have been attributed to inflammation and subsequent cytokine storm. Thromboelastography (TEG) is a point-of-care test used to assess clot formation and degradation in whole blood and is an indicator of the overall real-time coagulopathic state of the patient.

**Methods:**

A single-centre, prospective, observational cohort study was conducted in South Africa, analysing the coagulation patterns of 41 patients with hypoxia related to SARS-CoV-2 using serial thromboelastography (TEG) on admission, after 48 hours, and at resolution of hypoxia/day 10. Results: Two-thirds (*n* = 26) were women. The median age was 61 (IQR 50–67), and the majority (88%) were Black patients. Almost half (22) of the patients were critically ill and ventilated, with median SOFA and SAPS2 scores of 3 and 22 (IQR2-4 and 18–30), respectively. The prevalence of hypercoagulability was 0.54 (95% CI 0.46–0.62), whilst 29/41 (0.71, CI 0.64–0.78)) met the definition of hypofibrinolysis. Differences between the hypercoagulable (HC) and non-hypercoagulable groups remained apparent at 48 hours after anticoagulation. At this time point, the K time was significantly lower (*p* ˂ 0,01), and the *α*-angle (*p* ˂ 0,01) and maximum amplitude (MA) (*p* ˂ 0,01) were significantly higher in the HC cohort. At resolution of hypoxia, or day 10, only MA was significantly higher in the hypercoagulable group compared to the non-hypercoagulable group (*p* = 0.01). The initial impairment in fibrinolysis (Ly30), *α* angle, and MA were significantly associated with mortality, with *p* values of 0.006, 0.031, and 0.04, respectively.

**Conclusions:**

In this South African population, hypercoagulability was a highly prevalent phenomenon in COVID-19 disease. It was typified by hypofibrinolysis and a persistently elevated MA, despite anticoagulation therapy.

## 1. Introduction

Severe acute respiratory syndrome coronavirus 2 (SARS-CoV-2) causes coronavirus disease 2019 (COVID-19), a multisystem disease, which is typified by hypoxia, inflammation, increased microthrombosis and macrothrombosis [1–4], and fibrinolysis shutdown [5, 6]. HC has been characterised using various viscoelastic tests (VETs), specifically thromboelastography (TEG).

Severe COVID-19 is associated with coagulation abnormalities, specifically a raised D-dimer and an increased fibrinogen, which correlates with a worse prognosis [7] and an increased risk of thrombotic events [7–9], and is not consistent with a disseminated intravascular coagulopathy (DIC) [9–11] or a consumptive process. [7] The aetiology of hypercoagulability may be linked to endothelial inflammation, neutrophil extracellular traps, and increased platelet activity with increased microvesicles. [11, 12].

Currently, there is no gold standard definition of hypercoagulability as defined by TEG parameters, but HC is typified by a low Ly30 < 1%, a MA > 69 mm, an *α*-angle of >77°, R-time of < 4.3 minutes, and a K-time of < 0.8 minutes [9, 11, 13–15]. These findings demonstrate a decreased clot formation time, an increased clot strength, and impairment of the fibrinolytic system. Variations in the definition of HC include combinations of abnormalities in MA, clot formation time, and *α*-angle. [5, 7, 10, 15, 16] This COVID-19-associated state of hypercoagulability and impaired fibrinolysis has been associated with an increased risk of thromboembolism [5, 7, 11] and thrombotic events, respectively. [6].

There have been several studies [6, 7, 10, 15–17] assessing TEG abnormalities at a single time point and even fewer at another point during COVID-19 infection. [8, 9, 11].

Using a robust definition that included one hypercoagulant/procoagulant parameter in addition to impaired lysis, we sought to determine an accurate estimate of the prevalence of COVID-19-associated hypercoagulability. In addition, we performed two further TEG assessments to describe the dynamic changes after 48 hours of anticoagulation and at resolution/day 10 of hypoxemia. To our knowledge, this is the first study in the South African population.

## 2. Methods

### 2.1. Study Design and Site

This was a prospective, observational, cohort study of COVID-19 adult patients in a South African hospital.

### 2.2. Study Population, Inclusion, and Exclusion Criteria

All patients aged 18 years and older who tested positive for the SARS-CoV-2 using polymerase chain reaction (PCR) assay and were admitted between November 2021 and March 2021 were considered for enrolment and were sequentially approached during weekday office hours. Patients were included if they were expected to survive for longer than 48 hours and met the criteria for severe disease or critical illness. Severe disease was defined as oxygen saturation ≤ 92% with a respiratory rate ≥ 25 and, therefore, requiring supplemental oxygen support without the need for invasive or noninvasive ventilation. Critical illness was defined as hypoxemia and the need for additional ventilatory support, in the form of noninvasive or invasive ventilation. We excluded patients if they were pregnant, on oestrogen replacement therapy, and chronic anticoagulation therapy, including, but not limited to, aspirin, warfarin, clopidogrel, heparin, enoxaparin, and direct-acting oral anticoagulants.

### 2.3. Procedure

All enrolled patients had blood collected into a citrated tube for coagulation assessment using thromboelastography with the TEG®6s, Haemonetics, Braintree, MA, USA, at three set time points during hospital admission:

- Time point 1 (day of admission, either before enoxaparin administration or at least four hours after enoxaparin administration)
- Time point 2 (48 hours, three hours postenoxaparin administration)
- Time point 3 (resolution of hypoxemia—off oxygen therapy, three hours postenoxaparin administration) or on day 10 of admission, whichever occurred first

Patient demographic, clinical, laboratory, and hospital survival data were extracted from clinical notes. The following scores were calculated from the clinical information: disseminated intravascular coagulation (DIC) score as per International Society on Thrombosis and Haemostasis (ISTH) criteria [18], sepsis-induced coagulopathy (SIC) score [19], simplified acute physiology score 2 (SAPS2) [20], and sequential organ failure assessment (SOFA) scores [21], at the above three time points unless the enrolled subject was discharged or demised before the defined time.

### 2.4. TEG®6s Methodology and Study Definitions [22]

The TEG is a point-of-care test, is used to assess the formation and degradation of a clot in whole blood, and demonstrates abnormalities in coagulation, which may not be apparent when using standard coagulation tests including prothrombin time (PT), activated partial thromboplastin time (aPTT), platelet number, and antithrombin. TEG®6s assays employ various activators, including kaolin, heparinase, and tissue factor. The reaction time (*R*) describes the time to initial fibrin formation and 2 mm amplitude (initiation phase) and informs about clotting factors. The kinetic (*K*) time is the time taken to achieve a given clot strength of 20 mm amplitude (amplification phase) and indicates the initial phase of fibrin cross-linking. The *α*-angle measures the speed of clot formation and assesses the thrombin burst (propagation phase). Both the *K*-time and *α*-angle are dependent on fibrinogen. The maximum amplitude (MA) indicates the maximum strength of the clot and depends on platelet function and the contribution of fibrin. The lysis time at 30 minutes (Ly30) indicates the amount of fibrinolysis within 30 minutes of MA. [22].

We used a strict study definition for hypercoagulability (HC) requiring the presence of both an abnormality of lysis (low Ly30) and at least one of the following: a reduced *R*-time, reduced *K*-time, elevated *α*-angle, or an elevated MA on admission.

### 2.5. Anticoagulation Protocol

Enoxaparin dosing was based on the COVID-19 hospital protocol. Therapeutic dosing was used when there was an increased risk of hypercoagulability based on an elevated *D-*dimer, in the face of critical illness and severe hypoxemia or in the presence of thromboembolic events. Alternatively, prophylactic enoxaparin was administered if patients were hypoxemic, had a normal D-dimer level, and had no contraindication to enoxaparin administration. TEGs were performed at admission before anticoagulation administration and, at time points 2 and 3, were performed 3–4 hours after the anticoagulation dose to standardise the effect of anticoagulation and measure antifactor 10 a. All patients received appropriate anticoagulation as per hospital protocol.

### 2.6. Outcome Measures

We applied the study definition of HC to the patient data to calculate the prevalence of HC. We described the trajectory of TEG parameters using data from admission, after 48 hours, and at resolution. We used the admission non-TEG characteristics to describe the hypercoagulable and non-hypercoagulable states. Lastly, we described the TEG and non-TEG characteristics and their association with mortality.

### 2.7. Statistical Analysis

Study data were collected and managed using REDCap® (Research Electronic Data Capture) electronic data capture tool hosted at the University of the Witwatersrand. Statistical analyses were performed using Statistica® version 13.3 (TIBCO Software Inc., USA). Continuous variables are expressed as median (interquartile range (IQR)), and proportions/percentages were used for categorical variables. Continuous data were compared using the Mann–Whitney *U* test, whilst proportions were compared using the chi-square test. A *p*-value < 0.05 was considered statistically significant. We based our sample size on the minimum number of 12 required per group as a rule of thumb for a pilot study. [23] Assuming a mortality rate of 40%, we required a minimum sample size of 20 per group.

### 2.8. Ethics Considerations

Approval was received from the University Human Research Ethics Committee (Medical), M200728. Written informed consent from the patient or patient surrogate was obtained as per local ethics committee guidelines.

## 3. Results

### 3.1. Demographics

A total of 41 patients with COVID-19 were included from 4 November 2020 and 23 March 2021. All patients (41) had a TEG on admission, 38 had a second (at 48 hours after admission), and 25 had a third (either on day ten if still hypoxemic or at the resolution of hypoxemia, whichever came first). Two-thirds (*n* = 26, 63%) were women. The median age was 61 years (IQR 50–67). The majority (88%) were Black patients. The median SAPS II score was 22 (IQR 18–30).

Almost half (22/41, 54%) of the patients were critically ill and ventilated, and 19 were classified as “severe disease” (not ventilated but hypoxic and requiring supplemental oxygen therapy). On the day of admission, the median SOFA score was 3 (IQR 2–4), ISTH DIC score was 2 (IQR 0–2), and median SIC score was 2 (IQR 2–3). Half (54%) of enrolled patients had coexistent hypertension, 41% had diabetes mellitus, 39% had human immunodeficiency virus (HIV), and 15% were obese.

Overall, 20 of 39 (51%) patients were placed on therapeutic anticoagulation, and 19 of 39 (49%) were placed on prophylactic doses of enoxaparin. There were no differences in the proportion of patients on therapeutic anticoagulation between the HC and non-HC group, *X* [2] = 0.24, *p* = 0.62.

### 3.2. Prevalence

Twenty-two of the 41 (54%) patients met the study definition of hypercoagulability (HC) with a prevalence of 0.54 (95% CI 0.46–0.62), whilst 32/41 (prevalence of 0.78, CI 0.72–0.85) met the definition of hypofibrinolysis with Ly30 < 1%. Using a Ly30 threshold of ≤0.1%, the prevalence was 0.71 (CI 0.64–0.78).

### 3.3. Trajectory of TEG Parameters from Admission to Resolution

**Figure 1:**
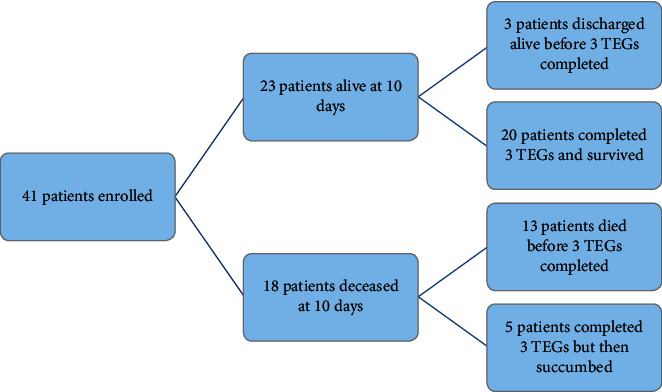
Flow diagram of a number of TEGs completed.

Hypercoagulability was defined by admission (T1) TEG parameters. Differences between the hypercoagulable and non-hypercoagulable groups become more apparent at 48 hours implying that there is a progression of the HC state, which only starts resolving after at least 48 hours. Although the *α*-angle and MA trended higher at baseline, these differences did not reach a significant statistical difference until 48 hours later. At 48 hours, the K-time is significantly lower (*p*˂0.01the *α*-angle (*p*˂0.01) and MA (*p*˂0.01) is significantly higher in the HC cohort. At resolution of hypoxia, or day 10, only MA was significantly and persistently higher in the hypercoagulable group compared to the non-hypercoagulable group (*p* = 0.01). As shown in Table 1 and Figure 1, this difference was driven by survivors (80%). At both 48 hours and resolution, median anti-Xa levels were within the range for anticoagulation prophylaxis, 0.42 (0.21–0.52) and 0.4 (0.27–0.84), respectively, within the HC group, and 0.42 (0.21–0.59) and 0.37 (0.26–0.7) in the non-HC group.

### 3.4. Hypercoagulable and Non-Hypercoagulable Groups

As shown in Table 2, platelet count at admission was significantly higher in the hypercoagulable group. There was also a trend to a lower haemoglobin level in the HC.

### 3.5. Mortality

Admission SOFA score, SAPS 2 score, and LDH were significantly higher amongst nonsurvivors. The initial impairment in fibrinolysis (Ly30), *α*-angle, and MA were significantly associated with mortality. As shown in Table 3, there was a trend to a higher D-dimer value amongst nonsurvivors.

## 4. Discussion

Severe COVID-19 is associated with coagulation abnormalities, known as CAC, specifically a raised D-dimer, which correlates with a worse prognosis [7] and an increased risk of thrombotic events. [14] The coagulation abnormalities are considered to be a hypercoagulable state typified by increased fibrinogen and increased D-dimers [7–9] and are not consistent with a disseminated intravascular coagulopathy (DIC) [9–11] or a consumptive process. [7] The aetiology of hypercoagulability may be linked to endothelial inflammation, neutrophil extracellular traps, and increased platelet activity with increased microvesicles. [11, 12].

Whilst traditional coagulation tests have been useful in the measurement of CAC, TEG is a point-of-care test used to assess the clot formation and degradation in whole blood in real time and can identify the individual contributions of the endothelium, the platelets, and the clotting factors. The fibrinolysis shutdown, described by Tsantes et al. [15] as maximum Ly30 < 3.5%, cannot be appreciated using conventional coagulation parameters. There have been several studies [15] looking at predominantly single-time point TEG assessments in critically ill patients with COVID-19, with some [8] having a repeat TEG, but for the first time, a third TEG assay was performed in this study of serial TEGs in patients with COVID-19, at time of resolution of hypoxia or day ten of illness.

The main finding was that half of the study population (54%) met the study definition of hypercoagulable state on their initial TEG, and 71% had a Ly30 ≤ 0.1%. No patients met the ISTH DIC criteria, with a mean DIC score of 2, and a mean SIC score of 2. The range of HC documented in studies has been variable. Considering clot lysis time alone as a marker for hypofibrinolysis, Bocci et al. found a Ly30 time of 0% on initial TEG in all 40 (100%) of their cohort [8], whilst Blasi et al. described “no lysis” in only 13% of the COVID-19 cohort. [10] A recent systematic review found that all the eligible studies had a Ly30 < 1% and three studies had a Ly30 of 0%. This study used a threshold for Ly30 of less than or equal to 0.1%, and our data showed a high prevalence (71%) of hypofibrinolysis [14].

Hypofibrinolysis on its own is an insufficient marker of abnormal coagulation, and at least one other TEG marker indicating increased clot burden may be found in 20% of “healthy” individuals. [24] To ensure robustness, our definition of hypercoagulability required both a lysis abnormality and one other TEG abnormality. The prevalence of COVID-19-associated HC ranges from 30% [16] (considering either an increased MA, K-time, or *α*-angle) to 74% [25] when only a MA > 69 mm is considered. Our proposed definition yields a prevalence of 54%, which may be less prone to overestimation and underestimation.

Aside from the absence of a gold standard definition, the timing of the viscoelastic test (VET) is likely to impact prevalence estimates. This is evident with studies conducting VET up to two weeks from admission. [26, 27] We chose to define HC based on admission TEG parameters to avoid the effects of anticoagulation. There may be additional heterogeneity based on different commercially available VET manufacturers (ROTEM, TEG, Quantra, and ClotPro) and differences in the patient population. Whilst our study provides the first data from South Africa in a predominantly Black population (88%), the effect of different analysers requires further investigation.

Despite similar anticoagulation strategies with similar proportional utilisation of prophylactic and therapeutic anticoagulation in the two groups, the HC group showed a significantly greater persistence of a functional procoagulant state over time. This is demonstrated with the hypercoagulable group demonstrating a shorter amplification (K-time) and greater propagation (higher *α*-angle) at 48 hours compared to the non-hypercoagulable group. There are no other data that we could find in the literature to compare these findings with. Both these processes normalise compared to the non-HC group by day 10/resolution. Two factors can be postulated to contribute to this change. The first is likely the effect of the administered anticoagulant, and the second may be the improvement of the HC state with disease resolution over time. After the administration of heparin in both groups, there was a nonstatistical increase in *R*-time between admission (preheparin) and 48 hours after admission (3 hours after the fourth dose of heparin). It is difficult to interpret the effect of heparin on the *R*-time as we used a heparinase-corrected reading, which may attenuate the increase in *R*-time.

The evolution of the functional coagulation variable relating to maximal clot strength (MA) is of great interest. The MA was significantly elevated and greater in the HC group when compared to the non-HC group. This elevation was present at 48 hours and persisted to resolution and/or day 10. This was found despite similar anticoagulation strategies and efficacy (anti-Xa levels). This difference was driven by the findings in the survivors (80% of those with TEGs at T3). Bocci et al. [8] described a similar persistence of TEG abnormalities at seven days despite therapeutic-dose anticoagulation.

Notably, 80% of clot strength and integrity are dependent on platelet function. [22] Our intervention included full anticoagulation for roughly half of the hypercoagulable group, whilst Bocci et al. used full anticoagulation for the entire group. The delayed persistence of this elevation may indicate that even therapeutic anticoagulation may not be able to completely reverse this hypercoagulable state.

Consideration should be given to the role of platelet aggregation, platelet hyperactivity, and microthrombus formation as these may be a neglected part of the pathophysiologic process of severe COVID-19. [28, 29] The MA represents the eventual strength of the fibrin clot and is not only predominantly dependent on platelet count in non-COVID-19 patients, interacting via GPIIb/IIIa, but also on fibrin. Fibrin has been thought to be the dominant role player in clot strength in patients with COVID-19. [30] The persistently elevated MA despite the resolution of hypoxia results in the continued stimulation of platelet activity in the damaged endothelium, particularly of the lungs, and the role of antiplatelet therapy may require further investigation within this cohort of patients.

The mechanism of platelet abnormality is multifactorial. There are HC and endothelialitis that occur with SARS-Co-V-2 infection, exacerbated by hypoxia (itself a platelet activator), and the milieu becomes procoagulant and proinflammatory. [2] Neutrophil extracellular traps may also play a role in initiating coagulation [2], as may an autoimmune component to the thrombocytopenia triggered by SARS-Co-V-2, or direct infection of the haemopoietic cells, as was seen in SARS, a similar coronavirus.

There have also been studies showing a normal [9] or increased platelet count [11], and possible causes include hypoxia with stimulation of hypoxia-inducible factor and increased megakaryocyte activity within the lung and bone marrow. [31] Furthermore, the spike protein of SARS-Co-V-2 can directly stimulate platelets. [32] Our own findings suggest an association between a higher platelet count and this state.

Hranjec et al. used platelet mapping and VET to guide the treatment of patients with COVID-19, and they corrected the platelet-induced aspect of the coagulopathy with either aspirin or clopidogrel or both (in addition to heparin), which was associated with improved outcomes. Viecca et al. performed a proof-of-concept case-control study and showed that patients treated with acetylsalicylic acid and clopidogrel had a reduction in alveolar to arterial oxygen gradient, consistent over seven days, with a concomitant increase in PaO2/FiO2 ratio, with no bleeding incidences. [33].

The fibrinolysis shutdown (Ly30) in our study was significantly lower in the nonsurvivors. This has been previously described, with Wright et al. [6] demonstrating a correlation between fibrinolysis shutdown and thrombotic events, and Bocci et al. [8] demonstrated a worse 28-day outcome within the group who had Ly30 < 1% and an elevated D-dimer, and an association between HC and rate of thrombotic events (TEs) and renal failure [6, 11, 34] amongst patients with COVID-19.

The median *α*-angle (clot propagation) was approximately 76° in nonsurvivors and 78° in survivors. Although this was statistically different, it is unlikely that these are clinically different as they both hover around the upper reference limit and the coefficient of variation of the assay is certainly larger than this difference. A similar relationship between MA and mortality is present. Both survivors and nonsurvivors have an elevated MA, but the absolute difference (0.8 mm) is unlikely to be clinically important. It is likely that we should, therefore, consider hypercoagulability as a continuum of effect, and larger studies may be required to establish clinical thresholds.

The combination of hypofibrinolysis and persistently increased MA alludes to the as yet not fully understood COVID-19 coagulopathy but suggests that platelets should not be overlooked and speaks to the complex nature of the disease process.

### 4.1. Limitations

This study was limited by its observational nature. Furthermore, it was a small study, at a single centre, and thromboembolic events were not assessed.

## 5. Conclusions

In this South African population, hypercoagulability is a highly prevalent phenomenon in COVID-19 disease. It is typified by hypofibrinolysis and a persistently elevated MA, despite anticoagulation therapy.

## Figures and Tables

**Table 1:** Changes in the TEG and anti-Xa levels between admission and at day 10/resolution.

TEG CKH	*T*1		*T*2		*T*3	
	*n*	Median (IQR)	n	Median (IQR)	*n*	Median (IQR)
*Hypercoagulable*						
LY30-CK (%)	22	0 [0–0.1]	20	0 [0–0]	16	0 [0–0]
TEG-ACT (sec]	22	83.2 [78.5–87.9]	20	92.6 [78.5–106.6]	16	92.6 [73.9–106.6]
*R*-time	22	4.6 [4–5.2]	20	5 [4.6–6.2]	16	4.5 [3.7–6]
*K*-time	22	0.8 [0.8–1.0]	20	**0.8 [0.8-0.9]** ^*∗*^	16	0.8 [0.8–1.1]
*α* angle	22	78.2 [76.9–79]	20	**79.4 [77.6-81.1]** ^*∗*^	16	77.5 [75.6–79.7]
MA	22	69.5 [68.8–70.4]	20	**70.3 [68.7-71.8]** ^*∗*^	16	**70 [68.9-72.3]** ^*∗*^

*Non-hypercoagulable*						
LY30-CK (%]	19	0 [0–0]	17	0 [0–0]	9	0 [0–0]
TEG-ACT (sec]	19	97.3 [78.5–116]	18	97.3 [87.9–116]	9	97.3 [87.9–106.6]
*R*-time	19	4.6 [3.5–5.3]	18	5.4 [4.9–6.1]	9	4.7 [4.3–5.9]
*K*-time	19	1 [0.8–1.8]	18	**1.3 [1-1.6]** ^*∗*^	9	1 [0.8–1.3]
*α*-Angle	19	75.4 [69.9–77.5]	18	**72.8 [71.5-76.9]** ^*∗*^	9	77.1 [72.7–79.7]
MA	19	65.3 [53.4–68.9]	18	**64 [58-65.7]** ^*∗*^	9	**65.6 [61.6-68.6]** ^*∗*^

^*∗*^Statistically significant. Data are expressed as median (interquartile range). *T*1 (admission), T2 (48 hours), *T*3 (resolution of hypoxia/day 10), TEG thromboelastography, ACT activated clotting time, CKH citrated kaolin heparinase, *R* reaction, K kinetics, MA maximum amplitude, LY30 lysis at 30 minutes.

**Table 2:** Non-TEG characteristics.

Variables	All patients, *n* = 41	HC, *n* = 22	Non-HC, *n* = 19	*p* value
Age (year)	61 [50–67]	62.5 [51–68]	56 [46–64]	0.22
Female, *n* (%)	26 (63)	14/42 (34%)	12/41 (29%)	

*Blood parameters*				
Haemoglobin (g/dL)	13.2 [11.6–14.2]	12.05 [10.6–14.00]	13.3 [13.00–15.00]	0.06
LDH (U/L)	752 [489–901]	759 [495–842]	651 [489–989]	0.98
D-dimers (mg/L)	0.585 [0.38–1.39]	0.58 [0.38–1.0]	0.96 [0.39–2.12]	0.45
Fibrinogen (mg/dL)	6.7 [5.6–7.8]	6.7 [6–7.9]	6.5 [4.9–7.3]	0.40
Platelets (x 10^9^/L)	249 [195–292]	265 [237–342]	221 [167–280]	0.02^*∗*^
PT (sec)	13.45 [13.0–14.4]	13.9 [13.0–14.4]	13.4 [12.9–14.5]	0.75
aPTT (sec)	25.6 [22.35–30.7]	24.7 [21.9–28.7]	25.85 [23.2–32.0]	0.60
INR	1.11 [1.06–1.19]	1.13 [1.06–1.18]	1.11 [1.06–1.2]	0.90

Enoxaparin, n = **39**	*n* = 39	*n* = 21	*n* = 18	0.62
Therapeutic, *n* (%)	20 (51)	10 (48)	10 (56)	
Prophylactic *n* (%)	19 (49)	11 (52)	8 (44)	

*Severity*				
Severe	19 (46)	10 (53)	9 (47)	
Critically ill	22 (54)	12 (55)	10 (45)	0.9
SOFA	3 [2–4]	3 [2–4]	2 [2–5]	0.49
SAPS2	22 [18–30]	26 [21–31]	21 [18–27]	0.13
DIC	2 [0–2]	2 [0–2]	2 [0–3]	0.16
SIC	2 [2–3]	2 [2–3]	2 [2–3]	0.87

Mortality, n (%)	18 (43.9)	8 (44.4)	10 (55.6)	0.30
Resolution of hypoxemia by day 10, n (%)	13 (32)	8 (36)	5 (26)	0.49

^*∗*^Statistically significant. Data are expressed as median (interquartile range) or count (percentage). HC hypercoagulable, DM diabetes mellitus, LDH lactate dehydrogenase, PT prothrombin time, aPTT activated partial thromboplastin time, INR international normalised ratio, SOFA sequential organ failure assessment, SAPS2 simplified acute physiology score 2, DIC disseminated intravascular coagulopathy, SIC sepsis-induced coagulopathy.

**Table 3:** Factors associated with mortality.

Initial variable	Survivors		Nonsurvivors		*p*-value
	Valid n	Median (IQR)	Valid n	Median (IQR)	
SOFA score	23	2 [2–3]	18	4 [2–6]	0.017
SAPS2 score	23	20 [18–26]	18	28 [21–43]	0.006^*∗*^
DIC score	23	2 [0–2]	18	2 [0–3]	0.112
SIC score	23	2 [2–3]	18	2 [2–3]	0.490
Platelets *x* 10 *∗* 9/L	23	269 [244–328]	18	196 [167–264]	0.004^*∗*^
Haemoglobin g/dL	23	13.3 [11.6–14.2]	18	13.2 [11.3–14.3]	0.765
LDH u/L	15	513 [413–757]	12	889 [387–1051]	0.003^*∗*^
Fibrinogen mg/dL	15	6.7 [6.0–7.5]	12	6.5 [5.35–7.95]	0.980
D-dimers mg/L	21	0.56 [0.34–0.97]	17	0.96 [0.39–2.12]	0.089
INR	22	1.11 [1.05–1.19]	17	1.13 [1.07–1.18]	0.440
PT sec	21	13.4 [12.8–14.2]	17	13.5 [13.1–14.4]	0.601
aPTT sec	21	25.2 [21.9–26.5]	15	29.9 [22.8–32.6]	0.141
LY30-CK (%)	23	0.1 [0.00–2.00]	18	0 .0 [0.0–0.0]	0.006^*∗*^
R–CKH	23	4.9 [4.2–5.5]	18	4.0 [3.4–5.2]	0.138
K–CKH	23	0.8 [0.8–1.0]	18	1.0 [0.8–1.8]	0.081
Ang-CKH	23	78.2 [75.3–79.6]	18	75.95 [69.90–78.10]	0.031^*∗*^
MA-CKH	23	69.1 [66.5–70.4]	18	68.3 [53.4–69.4]	0.040^*∗*^

^*∗*^Statistically significant. Data are expressed as median (interquartile range). LDH: lactate dehydrogenase, PT: prothrombin time, aPTT: activated partial thromboplastin time, INR: international normalised ratio, SOFA: sequential organ failure assessment, SAPS2: simplified acute physiology score 2, DIC: disseminated intravascular coagulopathy, SIC: sepsis-induced coagulopathy, TEG: thromboelastography, CKH: citrated kaolin heparinase, *R*: reaction, K: kinetics, MA: maximum amplitude, LY30: lysis at 30 minutes, Ang: angle, Anti-Xa: antifactor Xa.

## Data Availability

Anonymised supporting data will be made available upon request in an Excel spreadsheet.

## List of abbreviations used

anti-Xa: Anti-factor *X* a
aPTT: Activated partial thromboplastin time
CAC: COVID-19-associated coagulopathy
CHBAH SOP: Chris Hani Baragwanath Academic Hospital Standard Operating Procedure
CI: Confidence interval
CKH: Citrated kaolin heparinase
COVID-19: Coronavirus disease 2019
DIC: Disseminated intravascular coagulation
HC: Hypercoagulability
IQR: Interquartile range
ISTH DIC: International Society on Thrombosis and Haemostasis Disseminated Intravascular Coagulation score
K-time: Kinetic time
LDH: Lactate dehydrogenase
Ly(30): Lysis time at 30 minutes
MA: Maximal amplitude
PT: Prothrombin time
R-time: Reaction time
SARS-CoV-2: Severe acute respiratory syndrome coronavirus 2
SAPS2: Simplified acute physiology score 2
SD: Standard deviation
SIC: Sepsis-induced coagulopathy
SOFA: Sequential organ failure assessment
TE: Thrombotic events
TEG: Thromboelastography
TTP: Thrombotic thrombocytopenic purpura
VET: Viscoelastic test.
